# mRNA Expression and Role of PPARγ and PPARδ in Bovine Preimplantation Embryos Depending on the Quality and Developmental Stage

**DOI:** 10.3390/ani10122358

**Published:** 2020-12-10

**Authors:** Katarzyna Suwik, Emilia Sinderewicz, Dorota Boruszewska, Ilona Kowalczyk-Zięba, Joanna Staszkiewicz-Chodor, Krzysztof Łukaszuk, Izabela Wocławek-Potocka

**Affiliations:** 1 Department of Gamete and Embryo Biology, Institute of Animal Reproduction and Food Research, Polish Academy of Sciences, 10-747 Olsztyn, Poland; k.grycmacher@pan.olsztyn.pl (K.S.); e.sinderewicz@pan.olsztyn.pl (E.S.); d.boruszewska@pan.olsztyn.pl (D.B.); i.kowalczyk@pan.olsztyn.pl (I.K.-Z.); j.staszkiewicz-chodor@pan.olsztyn.pl (J.S.-C.); 2 Department of Obstetrics and Gynecological Nursing, Faculty of Health Sciences, Medical University of Gdansk, 80-210 Gdansk, Poland; luka@gumed.edu.pl; 3 Department of Obstetrics and Gynecology, The Medical Center of Postgraduate Education, 02-091 Warsaw, Poland; 4 INVICTA Fertility and Reproductive Center, 80-850 Gdansk, Poland

**Keywords:** embryos, ppar receptors, bovine

## Abstract

**Simple Summary:**

The results of the presented study proved that in vitro produced early- and late- cleaved bovine embryos express mRNA of peroxisome proliferator-activated receptor δ (PPARδ) and peroxisome proliferator-activated receptor γ (PPARγ) at all stages of early development (2-, 4-, 16-cell embryo, morula, blastocyst). The expression of PPARδ and PPARγ was correlated with the expression of quality markers in bovine blastocysts [sex-determining region Y-box 2 (SOX2), octamer-binding transcription factor 4 (OCT4), placenta-specific 8 (PLAC8), insulin-like growth receptor (IGF1R)]. It was found that in the group of early-cleaved embryos, which is after about 30 h after fertilization, positive correlations were stronger and more frequent, whereas the negative correlations were typical for group of late-cleaved embryos, which have a first cleave after 36 h.

**Abstract:**

Peroxisome proliferator-activated receptors (PPARs), a nuclear receptors for prostacyclin (PGI_2_) have been recognized as being essential for early embryo development. The objectives of the present study were to determine if the bovine early- and late-cleaved embryos in different stages of early development express PPARγ and PPARδ. Since embryo developmental competence depends on numerous biological factors, we evaluated if the expression of PPARγ and PPARδ correlate with selected embryo quality markers (SOX2, OCT4, PLAC8, IGF1R) in the in vitro produced embryos at different stages of their development. Developmental rates and embryo quality for early- and late-cleaved embryos were provided according to International Embryo Transfer Society (IETS; developmental stages: 2-, 4-, 16-cell embryo, morula, blastocyst (1—early, 2—developing, 3—expanded, 4—hatched); quality stages: A—high quality, B—moderate quality, C—low quality). We found that bovine embryos expressed mRNA of PPARδ and PPARγ at all stages of early development, independently of their quality. In addition, the expression of PPARδ and PPARγ correlated with the expression of quality markers in bovine blastocysts. Positive correlations were stronger and more frequent in the group of early-cleaved embryos, whereas the negative correlations were typical for the group of late-cleaved embryos. Obtained results and available literature reports may indicate the participation of PGI_2_, via PPARδ and PPARγ, in the processes related to the early embryo development, through the participation of this factor in the modulation of blastocyst hatching, implantation, and post-implantation development.

## 1. Introduction

Prostacyclin (prostaglandin (PG)I_2_, PGI_2_) belongs to the group of prostanoids, which are lipid mediators of the pathological processes, but also essential for the physiology of the female reproduction [1]. PGI_2_ is essential for pregnancy and early embryo development, including embryo hatching, blastocyst spacing, implantation, and decidualization [2,3,4]. It was documented that PGI_2_ is the most abundant PG produced by mouse blastocysts, acting as an antiapoptotic factor during early embryo development [4,5]. Moreover, expression of PGI_2_ receptors is known to be essential in the embryo development in human, rodents, and ruminants. [6,7,8]. Prostacyclin is produced by prostaglandin I_2_ synthase (PGIS) [9] and acts through two types of receptors: G-protein-coupled receptor (PTGIR), which induces an increase of intracellular cAMP mediating PGI_2_ vascular actions and nuclear peroxisome proliferator-activated receptors (PPARs), where the effect of PGI_2_ action is dependent on the type of the receptor [10,11,12]. There are three known isoforms of PPARs: PPARα, PPARγ, and PPARδ. PPARs are members of the nuclear receptor superfamily of transcription factors, whose action can be induced by a number of natural compounds such as fatty acids and eicosanoids, hypolipidemic agents and the thiazolidinedione class of antidiabetic drugs [11]. PPARs are activated by heterodimerization with retinoid X receptors (RXR) to form a PPAR/RXR complex. After that, PPAR-RXR heterodimers with the participation of a variety of transcriptional coactivator proteins necessary to activate transcription, bind to a specific DNA sequence called a PPAR-response element (PPREs) of the specific target genes [11,13]. Ligand-dependent activation of PPARs regulates the transcription of the intracrine nuclear pathways engaged in the control of energy, lipid metabolism, cell proliferation, and differentiation [11,13,14].

The presence of PPARδ in the trophectoderm is relevant for the PGI_2_ effect on blastocyst hatching [6]. Deficiency of PPARγ in murine embryos shortly after fertilization led to disturbances in the terminal differentiation of the vascularization in the placenta, and finally to the death of the embryo [7]. It has been shown that in cows, PPARγ mRNA is expressed in all stages of the embryonic development from the oocyte to the blastocyst [8]. Although there is some data in the literature about the prostanoid signaling pathway in the bovine reproductive tract, there is still lack of knowledge about the existence of the receptors for prostacyclin in the bovine preimplantation embryos, resulting in the possibility of PGI_2_ action during in vitro embryo development.

On the other hand, embryo developmental competence is associated with the expression of the number of biological factors, including maternal mRNAs and proteins, accumulated during oogenesis, which are known as embryo quality markers [15,16,17,18,19,20,21]. Among them are transcription factors such as octamer-binding transcription factor 4 (OCT4) and sex-determining region Y-box 2 (SOX2) [7,8], regulating cell differentiation and pluripotency, insulin-like growth factors (IGFs) and its receptors (IGF1R and IGF2R), which improve morphology and growth potential of embryos [17,18] and placenta-specific 8 (PLAC8), which is up-regulated in hatched compared to early blastocysts [19]. Moreover, our last study (in the publication process) showed that the expression of SOX2, OCT4, PLAC8, IGF1R, and IGF2R depends on the developmental stage of the embryo during the preimplantation development. Besides the above-mentioned parameters of the bovine embryo quality and embryonic developmental competences, there is also the timing of the first zygotic cleavage [20,21]. It has been documented that bovine zygotes, cleaved early after oocyte fertilization, are more feasible to develop to the blastocyst stage than their late-cleaving counterparts [22]. Based on the model of the time of the first cleavage, in this study we investigated if the bovine early and late cleaved embryos, being on different stages of early development, express PPARγ and PPARδ differently. Moreover, we evaluated if the expression of PPARγ and PPARδ correlated with embryo quality markers in the in vitro produced embryos at different stages of development.

## 2. Materials and Methods

### 2.1. Cumulus-oocyte Complexes (COCs) Collection

All experimental procedures were approved by the Local Animal Care and Use Committee in Olsztyn, Poland (Agreement No. 76/2014/DTN). Ovaries, irrespective of the stage of the estrous cycle, were collected from mature Holstein cows at a local abattoir and transported to the laboratory within 40 min in sterile 0.9% saline solution at 37 °C.

Cumulus-oocyte complexes (COCs) were isolated by aspiration from subordinate ovarian follicles, less than 5 mm in diameter. Using a stereomicroscope, COCs were searched and washed two times in Wash Medium (M199; #M5017, Sigma Aldrich, Munich, Germany) supplemented with 20 mM HEPES (#H4034 Sigma Aldrich, Germany), 25 mM sodium bicarbonate (#S4019 Sigma Aldrich, Munich, Germany), 0.4% bovine serum albumin (#A9418 BSA; Sigma Aldrich, Munich, Germany), and 40 µg/mL gentamicin (#G1272, Sigma, Aldrich, Munich, Germany). Only COCs comprising oocytes with homogeneous ooplasm and surrounded by at least three layers of compact cumulus cells were selected for the study.

### 2.2. In Vitro Maturation

Groups of 50 immature COCs were placed in four-well plates (#144444, Thermo Fisher Scientific, Waltham, MA, USA) containing 400 μL of maturation medium (TCM 199 Maturation Medium (19990/0010, Minitube, Tiefenbach, Germany) supplemented with 0.02 IU/mL pregnant mare’s serum gonadotropin (PMSG, #G4527, Sigma Aldrich, Munich, Germany), 0.01 IU/mL human chorionic gonadotropin (hCG, #C0684, Sigma Aldrich, Munich, Germany) and 5% fetal bovine serum (FBS, #12106C, Sigma Aldrich, Munich, Germany) and incubated at 38.5 °C in a 5% CO_2_ humidified air atmosphere for 24 h.

### 2.3. Semen Capacitation

For the in vitro fertilization, frozen-thawed semen from the same bull was used throughout the experiment. After thawing (proceeded in water bath, at 38 °C by 60 s), semen was layered below capacitation medium (TL sperm capacitation medium, 19990/0020, Minitube, Tiefenbach, Germany) supplemented with 1 mM sodium pyruvate, 0.6 % BSA and 0.1 mg/mL gentamicin) and incubated for 1 h at 38.5 °C in 5% CO_2_ humidified air atmosphere to allow the recovery of motile sperm through the swim-up procedure. Then, the upper two-thirds of capacitation medium was recovered, centrifuged at 200× *g* for 10 min, the supernatant was removed, and the sperm pellet was diluted in an appropriate volume of fertilization medium to give a final concentration of 10^6^ sperm/mL.

### 2.4. In Vitro Fertilization

After in vitro maturation, groups of 50 COCs were placed in fertilization medium (TL fertilization medium; 19990/0030, Minitube, Tiefenbach, Germany) supplemented with 10 µg/mL heparin (#H3393, Sigma Aldrich, Munich, Germany), 20 mM sodium pyruvate (#P3662, Sigma Aldrich, Munich, Germany) and 0.5 % BSA and co-incubated with spermatozoa in four-well dishes containing 400 μL of fertilization medium for 30 h at 38.5 °C in 5 % CO_2_ humidified air atmosphere. The day of in vitro insemination was considered Day 0. Following fertilization, embryos were separated from cumulus cells by vortexing and washed three times in wash medium. According to Patel et al. [21], for collection of early- and late-cleaved embryos, pools of early-cleaved two-cell embryos (*n* = 5; each pool consists of five embryos, for RNA extraction) were collected at 30 h post-fertilization. Pools of late-cleaved embryos (*n* = 5; each pool consists five embryos, for RNA extraction) were isolated 36 h post-fertilization.

### 2.5. In Vitro Embryo Culture

Early- and late-cleaved embryos were cultured separately in four-well dishes, containing 400 μL culture medium (SOF; synthetic oviduct fluid medium, SOF; 19990/0040, Minitube, Tiefenbach, Germany) supplemented with amino acids: 10 μL /mL BME (#B6766, Sigma Aldrich, Munich, Germany) and 20 μL/mL MEM (#M7145, Sigma Aldrich, Munich, Germany), 3.3 mM sodium pyruvate and 5% FBS under 400 μL of mineral oil at 38.5 °C in an atmosphere of 5 % CO_2_, 5 % O_2_, 95 % N_2_ with high humidity, until they reached blastocysts stage (Day 7).

Developmental rates and embryo quality for early- and late-cleaved embryos were provided by morphological examination on stereo- or inverted microscope at 20, 50 or 100× magnification, according to the International Embryo Transfer Society (IETS; International Embryo Transfer Society 1998; developmental stages: 2-, 4-, and 16-cell embryo, morula, blastocyst (1—early, 2—developing, 3—expanded, 4—hatched); quality stages: A-high quality, B-moderate quality, C-low quality).

### 2.6. Total RNA Extraction, Reverse Transcription (RT) and Real-Time PCR

For mRNA expression analysis, total RNA was extracted from five pools of embryos at different stages of their preimplantation development. Total RNA was extracted according to the Arcturus PicoPure RNA Isolation Kit protocol (#KIT0204, Applied Biosystems, Waltham, MA, USA). The RNA samples were stored at −80 °C. Before use, RNA content and quality were evaluated by spectrophotometric measurement. The amount of 500 ng of each sample of total RNA was reverse transcribed (according to Super Script III reverse transcriptase protocol (#18080-044, Invitrogen, Carlsbad, CA, USA). Products of RT were diluted with sterile water 5× to final concentration 10 ng/µL and stored at −20 °C until real-time PCR amplification.

The expression of mRNA for PPARγ, PPARδ, SOX2, OCT4, PLAC8, IGF1R, and IGF2R were measured by real-time PCR. Real-time PCR was performed with an ABI Prism 7900 (Applied Biosystems, Life Technologies, Waltham, MA, USA) sequence detection system using the Maxima Sybr Green/ROX qPCR Master Mix (#K0222, Thermo Fisher Scientific, Waltham, MA, USA). The polymerase chain reactions (PCR) were performed in 384-well plates. In each reaction, a quantity of cDNA equivalent to 0.15 blastocyst was used. The results of mRNA transcription were normalized to glyceraldehyde-3-phosphate dehydrogenase (GAPDH, an internal control) mRNA levels and were expressed as arbitrary units. The housekeeping gene was chosen using the NormFinder software by comparing the following candidate genes: GAPDH, β-actin and H2A.1 [23]. The primers were designed using an online software package [24]. The primer sequences and the sizes of the amplified fragments of all of the transcripts are shown in Table 1. For the relative quantification of mRNA levels, Miner software was used [25].

### 2.7. Statistical Analysis

**Figure 1:**
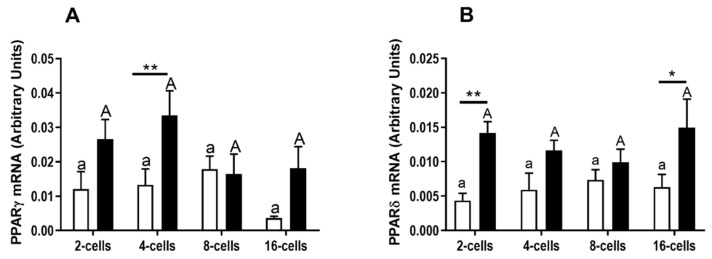
(**A**) The mRNA relative abundance of the PPARγ and (**B**) PPARδin bovine embryos at the early stages of development: 2-, 4-, 8-, and 16 cells, produced from early- (white bars) and late-cleaved zygotes (black bars). Small (a,b) and capital (A,B) superscript letters indicate differences in the respective mRNA ratios between the various developmental stages of embryos produced from early- and late-cleaved zygotes, respectively. Asterisks denote significant differences in mRNA ratios between the embryos produced from early- and late cleaved zygotes, within this same developmental stage. (* means *p* ≤ 0.05; ** mean *p* ≤ 0.01). Statistical analysis was determined by a two-way analysis of variance (ANOVA) followed by Bonferroni’s multiple comparison test (*p* < 0.05).

**Figure 2:**
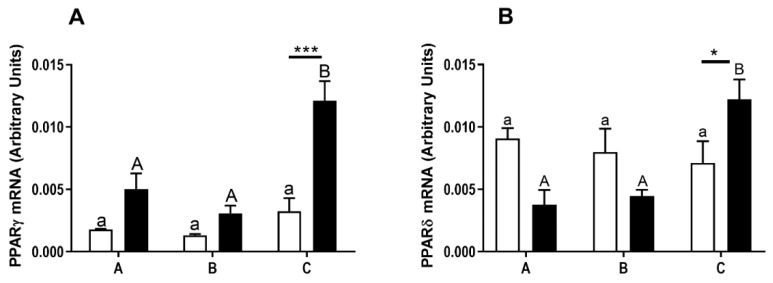
(**A**) The mRNA relative abundance of the PPARγ and (**B**) PPARδin varied in quality (A—high, B—moderate, C—low quality) bovine morulas produced from early- (white bars) and late-cleaved zygotes (black bars). Small (a,b) and capital (A,B) superscript letters indicate differences in the respective mRNA ratios between the various quality groups of morulas, produced from early- and late-cleaved zygotes, respectively. Asterix denotes significant differences in mRNA ratios between the morulas produced from early- and late cleaved zygotes, within the same quality group (* means *p* ≤ 0.05; *** mean *p* ≤ 0.001). Statistical analysis was determined by a two-way ANOVA followed by Bonferroni’s multiple comparison test (*p* < 0.05).

**Figure 3:**
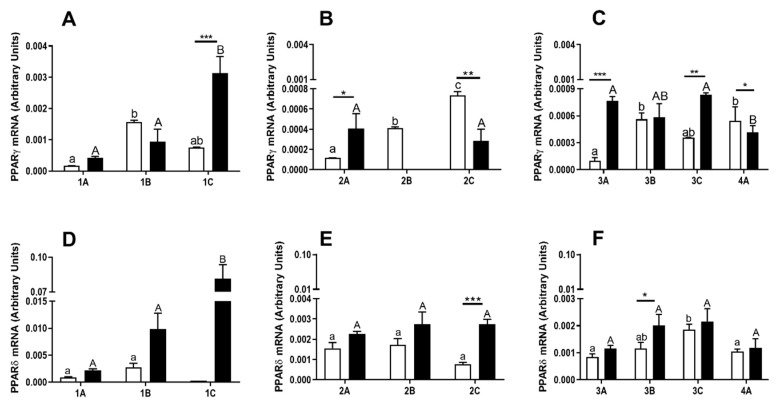
(**A**–**C**) The mRNA relative abundance of the PPARγ and (**D**–**F**) PPARδin varied in quality (A—high, B—moderate, C—low quality) and developmental stage bovine blastocysts (1—early blastocyst (Figure 3A,D), 2—developed blastocyst (Figure 3B,E), 3—expanded and 4—hatched blastocyst (Figure 3C,F) produced from early- (white bars) and late-cleaved zygotes (black bars). Small (a,b) and capital (A,B) superscript letters indicate differences in the respective mRNA ratios between the various quality groups of blastocysts, produced from early- and late-cleaved zygotes, respectively. Asterix denotes significant differences mRNA ratios between the blastocysts produced from early- and late cleaved zygotes, within this same developmental stage and quality group (* means *p* ≤ 0.05; ** mean *p* ≤ 0.01; *** mean *p* ≤ 0.001). Statistical analysis was determined by a two-way ANOVA followed by Bonferroni’s multiple comparison test (*p* < 0.05).

**Figure 4:**
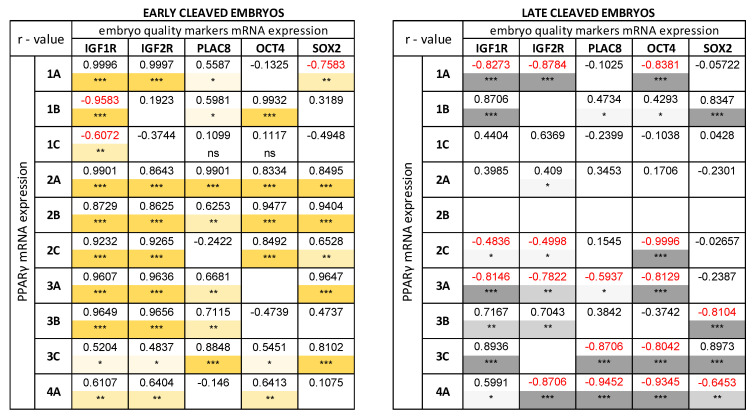
The correlation between PPARγ and embryo quality markers (IGF1R, IGF2R, PLAC8, OCT4, SOX2) mRNA expression in the bovine blastocyst-stage embryos developed from early-cleaved embryos (yellow table) and late-cleaved embryos (grey table). The numbers 1, 2, 3, 4 indicate developmental stage of blastocysts (early, developed, expanded, and hatched, respectively). The superscript letters A, B, C indicate quality of the blastocyst (high, moderate, and low, respectively). The white field indicates a non-significant r-value (r < 0.4). The *, **, and *** and color tonality indicate strength of the correlation (weak (0.41 < r < 0.6, light tonality), moderate (0.61 < r < 0.8, moderate tonality) and strong correlation (0.81 < r < 1.0, dark tonality), respectively). r-values of negative and statistically significant correlations were marked in red. The correlation analysis was followed by a Pearson test.

**Figure 5:**
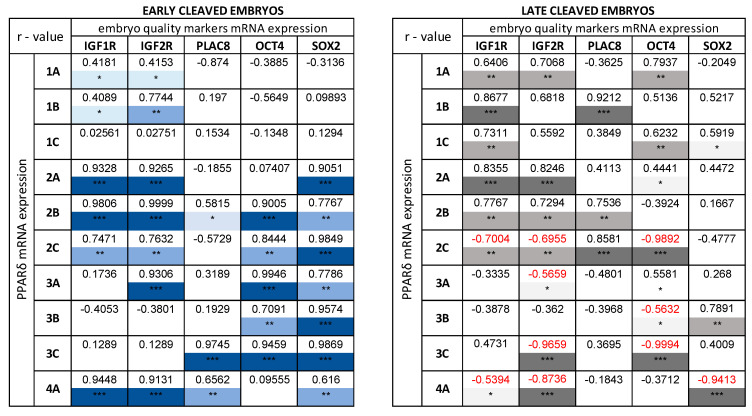
The correlation between PPARδ and embryo quality markers (IGF1R, IGF2R, PLAC8, OCT4, SOX2) mRNA expression in the bovine blastocyst-stage embryos developed from early-cleaved embryos (blue table) and late-cleaved embryos (grey table). The numbers 1, 2, 3, 4 indicate developmental stage of blastocyst (early, developed, expanded, and hatched, respectively). The superscript letters A, B, C indicate quality of the blastocyst (high, moderate, and low, respectively). The white field indicates a non-significant r-value (r < 0.4). The *, **, and *** indicate strength of correlation (weak (0.41 < r < 0.6), moderate (0.61 < r < 0.8) and strong correlation (0.81 < r < 1.0), respectively). r-values of negative, statistically significant correlations were marked in red. The correlation analysis was followed by a Pearson test.

Statistical analyses were conducted using GraphPad PRISM v. 6.0 software (GraphPad Software, Inc., La Jolla, CA, USA). All experimental data are shown as the mean ± SEM, and the differences were considered significantly different at the 95% confidence level (*p* < 0.05). The analyses were performed using ANOVA, followed by the Bonferroni’s multiple comparison test (Figures 1–3) or correlation analysis followed by Pearson’s test (Figures 4 and 5).

## 3. Results

### 3.1. The Expression of PPARγ and PPARδ in the Bovine Embryo at the Early Stages of Development

The expression levels of the mRNA of PPARγ (Figure 1A) and PPARδ (Figure 1B) were determined in the bovine early- and late-cleaved embryos at the early stages of development: 2-, 4-, 8-, and 16-cells. Similar mRNA expression of PPARγ (Figure 1A, *p* > 0.05) and PPARδ (Figure 1B, *p* > 0.05) was detected in all stages of early embryo development in early- (Figure 1A,B, white bars) and late-cleaved (Figure 1A,B, black bars) embryos. The expression of PPARγ mRNA was higher in the late-cleaved 4-cell embryos in comparison to the early-cleaved embryos of the same stage of development (Figure 1A, *p* < 0.05). The expression of PPARδ mRNA was higher in the late-cleaved 2-cells and 16-cells embryos in comparison to the early-cleaved embryos of the same stages of development (Figure 1B, *p* < 0.05).

### 3.2. Expression of PPARγ and PPARδ in the Bovine Morulaes

The expression levels of the mRNA of PPARγ (Figure 2A) and PPARδ (Figure 2B) were determined in the early- (Figure 2A,B, white bars) and late-cleaved (Figure 2A,B, black bars) embryos. The expression of PPARγ and PPARδ mRNA was higher in morulaes with the lowest quality, produced from the late-cleaved embryos (Figure 2A,B, *p* < 0.05), in comparison to high and moderate quality morulaes, whereas the expression of PPARγ and PPARδ mRNA was similar in morulaes obtained from early-cleaved embryos, independently from their quality (Figure 2A,B, *p* > 0.05). The expression of PPARγ and PPARδ mRNA in morulaes with the lowest quality was lower in embryos gained from early-cleaved embryos in comparison to embryos gained from late-cleaved embryos (Figure 2A,B, *p* < 0.05).

### 3.3. The Expression of PPARγ and PPARδ in the Bovine Blastocysts

The expression of PPARγ and PPARδ mRNA was determined in the early (Figure 3A,D, respectively), developing (Figure 3B,E, respectively), expanded and hatched blastocysts (Figure 3C,F, respectively), produced from early-cleaved (Figure 3, white bars) and late-cleaved embryos (Figure 3, black bars).

The expression of PPARγ mRNA depended on the quality of the blastocysts, gained from early- and late-cleaved embryos (Figure 3A–C, *p* < 0.05). The mRNA expression of PPARγ was the highest in the moderate-quality early blastocysts gained from early-cleaved embryos, and in the low-quality early blastocyst gained from late-cleaved embryos (Figure 3A, *p* < 0.05). In developed blastocyst gained from early-cleaved embryos, the lowest mRNA expression of PPARγ was detected in high quality blastocysts, whereas the greatest mRNA expression of PPARγ was detected in low-quality embryos (Figure 3B, *p* < 0.05). The mRNA expression of PPARγ in high- and low- quality developed blastocyst gained from late-cleaved embryos was similar. The expression of PPARγ in moderate-quality blastocysts was not detected (Figure 3B, *p* > 0.05). In expanded blastocysts gained from early-cleaved embryos, the mRNA expression of PPARγ was higher in moderate-quality blastocysts in comparison to high- and low- quality blastocysts (Figure 3C, *p* < 0.05). Expanded blastocysts obtained from late-cleaved embryos presented a similar PPARγ mRNA level in all examined classes of quality.

The mRNA expression of PPARγ differed also among early- and late-cleaved embryos at the blastocyst stage of development. The greater mRNA levels of PPARγ was documented in low-quality early (Figure 3A, *p* < 0.05) and expanded blastocysts (Figure 3B, *p* < 0.05), and in high-quality developed (Figure 3B, *p* < 0.05) and expanded blastocysts (Figure 3C, *p* < 0.05), gained from late-cleaved embryos in comparison to early-cleaved embryos at the equivalent stages of development. Moreover, the mRNA expression of PPARγ was higher in low-quality developed (Figure 3B, *p* < 0.05) and high-quality hatched blastocysts (Figure 3C, *p* < 0.05) gained from early-cleaved embryos in comparison to blastocysts in the same developmental stages, produced from late-cleaved embryos.

The mRNA expression of PPARδ was blastocyst quality-dependent in early blastocysts gained from late-cleaved embryos and in expanded blastocysts obtained from early-cleaved embryos (Figure 3D,F, *p* < 0.05). The greatest mRNA level of PPARδ was detected for low-quality blastocysts, whereas the lowest mRNA level was detected in high-quality blastocysts (Figure 3D,F, *p* < 0.05). The mRNA expression of PPARδ was higher in low-quality developing and moderate-quality expanded blastocysts produced from late-cleaved embryos in comparison to early-cleaved embryos at the same stages of development (Figure 3E,F, *p* < 0.05).

### 3.4. Correlation between mRNA Expression of PPARγ, PPARδ and Embryo Quality Markers (IGF1R, IGF2R, PLAC8, OCT4, SOX2) in the Bovine Blastocysts

The correlations between PPARγ (Figure 4), PPARδ (Figure 5) and embryo quality markers (IGF1R, IGF2R, PLAC8, OCT4, SOX2) have been studied for various quality and developmental stages blastocysts, produced from early- (Figure 4A and Figure 5A) and late-cleaved embryos (Figure 4B and Figure 5B). Exact values (Table S1) and *p*-values (Table S2) of mRNA expression of PPARγ and PPARδ, and embryos at different stages of development and quality are documented in Supplementary files. In the group of blastocysts produced from early-cleaved embryos, numerous positive and three negative correlations between PPARγ and embryo quality markers were found (Figure 4A). The most numerous and strongest relationships were observed for developing blastocysts, regardless of quality. The low-quality early and expanded blastocysts revealed weaker and fewer correlations between PPARγ and embryo quality markers mRNA expression in comparison to moderate- and high-quality blastocysts (Figure 4A). The relationships between PPARγ and IGF1R, IGF2R, PLAC8, OCT4 and SOX2 mRNA expression were also found in the group of blastocysts produced from late-cleaved embryos, irrespective of embryo quality and stage of development (Figure 4B). However, the general number of correlations was lower and number of negative correlations was higher in the group of blastocysts produced from late-cleaved embryos in comparison with the group of blastocysts received from early-cleaved embryos.

The great number of correlations between mRNA expression of PPARδ and embryo quality markers in bovine blastocysts were observed (Figure 5A,B). In the group of blastocysts gained from early-cleaved embryos, the observed correlations were exclusively positive (Figure 5A), whereas in blastocysts obtained from the late-cleaved embryos many negative relationships were noticed (Figure 5B). The greatest number of correlations occurred between PPARδ-SOX2 and PPARδ-IGF2R in the group of early-cleaved blastocysts, independently of blastocyst quality and developmental stage (Figure 5A).

## 4. Discussion

The PPARα and PPARγ were considered to be expressed during late embryonic development [26], until the study of Mohan et al. [8], which described PPARγ mRNA expression during early bovine embryo development from the oocyte to the blastocyst. However, this study is the first to show an expression of both PPARγ and PPARδ in the bovine preimplantation embryo, correlating with embryo quality markers at different stages of development. We have found the presence of PPARγ and PPARδ in 2-, 4-, 8-, and 16-cell bovine embryos, morulaes and early, developed, expanded, and hatched blastocysts, produced from high- and low-quality oocytes. Moreover, the expression of PPARs was dependent of the developmental stage of the embryos and their quality. The mRNA expression of PPARγ was greater in 4-cells embryos, whereas mRNA expression of PPARδ was greater in 2- and 16-cells embryos in comparison to the other embryos in early developmental stages, suggesting possible PGI_2_ influence on the bovine early embryo development. These findings are consistent with the study of Huang et al. [3], who demonstrated that the supplementation of the culture medium with the PGI_2_ agonist resulted in the enhanced blastocyst hatching. However, this effect was not achieved through the membrane G-protein-coupled receptors, which accounts for the role of PPARs in the extended blastocyst hatching rate. The elevated mRNA levels of the examined PPARs during 2- and 4-cell stages suggest possible PGI_2_ influence before the maternal genome transition during early embryo development. On the other hand, the increased mRNA abundance of PPARδ at 16-cell stage, when the embryo genome is activated, suggests that the probable inclusion of the PGI_2_/PPARδ pathway during embryo development is also under the embryo genome control.

In this study, we also detected the expression of PPARγ and PPARδ in morulas and blastocysts, dependently on their quality, determined according to IETS guidelines. We found that the expression of the examined PPARs was higher in low-quality morulas, produced from late-cleaved zygotes, which underwent the first embryonic division after 36 h—not after 30 h, as their early-cleaved counterparts. Similarly, only low quality early and developed blastocyst revealed differences between PPARs expression in groups produced from early- and late-cleaved zygotes. This might suggest the existence of some counterbalancing mechanisms, engaging PGI_2_ to support low quality embryos to grow and develop. On the other hand, PPARγ mRNA expression in hatched blastocysts was higher in embryos produced from early-cleaved zygotes. Taking into consideration that early cleaved bovine embryos have greater developmental competences [20,21], our results seem to be consistent with a study carried out on mice, demonstrating the role of PPARγ in the terminal embryo differentiation and placental vascularization, preventing embryonic lethality [7]. What is more, PPARγ was involved in the accumulation of lipids in trophoblast, its differentiation and invasion during early pregnancy as well as regulation of conceptuses elongation [26,27,28].

PPARδ is known as the omnipresent expressed receptor, playing an extensive role during early embryo development [29]. Accordingly, the mRNA expression profile of PPARδ documented in this study, is surprising. We have found the expression of PPARδ in all examined groups of embryos, but the higher expression of PPARδ was detected in the lowest quality morulaes and developing blastocysts, which were produced from late-cleaved embryos. On the other hand, the PPARδ, which was expressed higher in the bovine blastocysts in comparison to PPARγ, is known as the part of the PGI_2_/PPAR axis, participating in blastocyst hatching in vitro. The lack of the active PGI_2_-PPARδ system caused failures in blastocyst formation and hatching but did not impair blastocyst development [5,6,30]. Moreover, in rodents, PPARδ activation by PGI_2_ increased also the quality of blastocyst implantation sites, blastocyst spacing, and decidualization [2,31,32]. Although little was known about the PPARδ involvement in the embryonic development in farm animals, Guo et al. [31] has recently demonstrated that the activation of PPARδ facilitated blastocyst hatching via fatty acid oxidation in pigs. Besides, treatment of porcine parthenotes with the PPARδ-specific agonist improved blastocyst quality via increasing the total cell number, which suggests that PPARδ affected the developmental competences of the embryo [31]. The presence of PPARδ and its role during the peri-implantation period was also documented in the ovine trophoblast [32]. In addition, Roberti et al. [33] suggested that PPARδ was the key factor in post-implantation development, preventing embryo resorption. Taking the above into consideration, the presence of PPARs seems to be essential during bovine embryo development, but mechanisms of their regulatory function need to be proven in further studies. Special attention should be directed to the ability of PPARs to heterodimerization with RXRs, which mainly determines the possibility of DNA binding and activation of gene transcription [13]. It has been shown that mice lacking of RXRα, which is major RXR isoform in placenta, were shown similar lethal placental defects as Pparγ-null and Pparδ-null embryos [34]. The expression and action of co-activators, such as CBP or p300 factors, for which have been shown that deficiency caused embryo dying during early gestation mice, should also be taken into account [13].

Despite numerous documents, showing the presence of PPARs in embryonic development and its influence on the key events as blastocyst hatching, implantation and decidualization, the mechanisms of PPARs/PGI_2_ action are still unknown. The embryo developmental competences are reflected by the expression of biological factors, as well as the time of the embryo first cleavage. In this study, we also conducted analyses of potential correlations of the mRNA expression of PPARs and the mRNA expression of SOX2, IGF1R, IGF2R, OCT4, and PLAC8. We planned to establish whether the involvement of PPARs during bovine embryo development was related to the abundance of expression of the embryo developmental quality markers.

We have found many correlations between mRNA expression of examined PPARs and mRNA expression of SOX2, IGF1R, IGF2R, OCT4, PLAC8. Interestingly, negative correlations between mRNA expression of PPARγ and PPARδ and mRNA expression of chosen quality markers were typical only for blastocysts produced from late-cleaved embryos. The above data suggest, that the presence of PPARs in the late-cleaved blastocysts or/and in low-quality embryos, being at the earlier stages of development, is not an effect of the regulatory mechanism, leading to improvement of their developmental competences, but rather some constitutive characteristics during bovine embryo development. Moreover, negative correlations between the expression of PPARs and factors involved in the proliferation and differentiation indicate that PPARs are engaged in some kinds of mechanisms regulating pregnancy establishment depending on the quality and developmental competences of the embryo. The positive correlations were documented in both early- and late-cleaved groups of blastocysts. However, the general number of correlations was lower and weaker in the group of blastocysts produced from the late-cleaved embryos in comparison to early-cleaved ones. In addition, the most numerous and strongest relationships were observed between PPARγ and IGF1R, IGF2R, OCT4, and SOX2 in developing blastocysts, regardless of their quality.

The greatest number of correlations occurred between PPARδ–SOX2 and PPARδ–IGF2R in the group of early-cleaved blastocysts, independently on the blastocyst quality and developmental stages. The SOX2, together with OCT4, both belong to the transcription factors, affecting embryo development via controlling cell proliferation, pluripotency, and differentiation [15,35]. Embryos with reduced expression of SOX2 did not develop into blastocysts [16]. What is more, the lack of SOX2 in bovine zygotes decreased the blastocyst rates due to deficiency of maternal-embryonic genome transition [36]. Besides, in early bovine embryos, transcripts of SOX2 were also described in oocytes [37]. Positive correlations of PPARs and SOX2 mRNA expression, documented mostly in blastocysts produced from high quality oocytes, might be caused by the presence of the SOX2 during all the steps of embryo development. This particular strong and abundant correlation was observed between SOX2 and PPARδ expression, which is known as the factor playing a significant role in blastocyst hatching and embryo development in general [29]. Its relationship with SOX2 expression may indicate the importance of PPARδ during bovine embryo development. In mice, the lack of the OCT4 resulted in arresting the embryos at multi-cell stages, leading to the decreased blastocyst rate [38]. The expression of OCT4 was also greater in early-cleaved embryos in comparison to the late-cleaved embryos [39,40], which is a consequence of the reduced developmental competences of the low-quality oocytes. Similarly, in this study, we observed positive correlations between examined PPARs and OCT4 expression in blastocysts produced from early-cleaved zygotes, whereas in the group of blastocysts produced from late-cleaved zygotes were found multiple negative correlations. The dependence of OCT4 expression on the bovine embryo developmental stage and quality was also found earlier (own unpublished data). The described correlations of SOX2, OCT4, and PPARs, account for the possible, multifunctional role of PPARs during bovine early embryo development.

It has been shown before that IGF and its receptors, IGF1R and IGF2R are also interdependent with the growth potential of the embryos [17,18,41]. In cows, the expression of IGF1R and IGF2R was the greatest during embryonic genome activation and in the hatched blastocyst [42], indicating its early role after fertilization as well as during implantation and formation of filamentous conceptus. IGF1R and IGF2R were also differentially expressed during the subsequent developmental stages of bovine embryos (own unpublished data). In our study, we found strong, positive correlations between the expression of IGF2R and examined PPARs in the early-cleaved embryos in comparison to the group of late-cleaved zygotes, which indicates the role of PPARγ and PPARδ during the peri-implantation period of the bovine embryo development.

It was demonstrated that PLAC8 was also involved in the early embryo development. The presence of PLAC8 was found for different developmental stages of the bovine embryo (own unpublished data). Furthermore, the expression of the PLAC8 was up-regulated in expanded and hatched compared to early blastocysts [21]. The higher expression of PLAC8 was documented in the blastocysts, which resulted in calf delivery compared to the blastocysts which had been resorbed [43,44]. Although the role of PLAC8 during early bovine embryo development is already established, in this study we documented the lowest number of correlations between the expression of PLAC8 and PPARs in blastocysts, which suggests that these pathways, both involved in blastocyst development, might act independently.

## 5. Conclusions

In summary, mRNA of PPARδ and PPARγ were expressed in the bovine embryos at different stages of development, independently of their quality. The expression of PPARδ and PPARγ correlated with the expression of quality markers in bovine blastocysts. Positive correlations were stronger and more abundant in the group of early-cleaved embryos, whereas the negative correlations were typical for the group of late-cleaved embryos. According to the above results and literature reports, we suggest the role of PGI_2_, acting via PPARδ and PPARγ, in mechanisms regulating bovine early embryo development, presumably via the modulation of blastocyst hatching, implantation, and post-implantation development. For the thorough examination of the mentioned mechanisms, further studies are required.

The obtained data provided additional information on the preimplantation development of the bovine embryo. Differences in the expression of genes for prostacyclin receptors and genes that are markers of early embryonic development, as well as the presented correlations, can increase information on the production of in vitro embryo development, which is a frequently used method in assisted reproductive technology (ART). In addition, the obtained data on gene expression regarding the various developmental stages as well as the quality of the embryos influence, can be used in ART of dairy and beef cattle, where morphological assessment is one of the main tools for embryo assessment.

## Figures and Tables

**Table 1:** Sequences of PCR primers (F = forward, R = reverse) and GenBank codes for gene transcripts used to assess the relative mRNA abundance of PPARγ, PPARδ, OCT4, SOX2, PLAC8, IGF1R, IGF2R, and GAPDH in bovine embryos at different stages of development. Amplicon sizes are expressed in the number of base pairs (bp).

Gene	Gene Bank ID	Primer Sequences (3′-5′)	Amplicon Size (bp)
PPARγ	NC_037349.1	F: AGTGGAGACCGCCCAGGTTTGR: GGGAGGACTCGGGGTGGTTC	104
PPARδ	NM_001083636.1	F: TGCGGGGCTTCTCCCATGACR: AGCCGTGAGTCCTGCCAAGT	81
OCT4	NM_174580.2	F: GAGAAAGACGTGGTCCGAGTGR: GACCCAGCAGCCTCAAAATC	101
SOX2	NM_001105463.2	F: TGGATCGGCCAGAAGAGGAGR: CAGGCGAAGAATAATTTGGGGG	89
PLAC8	NM_001025325.2	F: TTTACCGCTCTGTGCCCTTTR: CCATGTGAACTTGACCAAGCAT	95
IGF1R	NM_001244612.1	F: GAGTGGAGAAATCTGCGGGR: AAATGAGCAGGATGTGGAGGT	110
IGF2R	NM_174352.2	F: ACCTCCGATCCTCAATCCCAR: TGTAGTTGAAGTGCCGGTCC	82
GAPDH	NM_001034034.2	F: CACCCTCAAGATTGTCAGCAR: GGTCATAAGTCCCTCCACGA	103

PPARγ—peroxisome proliferator-activated receptor γ; PPARδ—peroxisome proliferator-activated receptor δ; OCT4—octamer-binding transcription factor 4; SOX2—sex-determining region Y-box 2; PLAC8—placenta-specific 8; IGF1R—insulin like growth factor 1 receptor; IGF2R—insulin like growth factor 2 receptor; GAPDH—glyceraldehyde-3-phosphate dehydrogenase.

## Supplementary Materials

The following are available online at https://www.mdpi.com/2076-2615/10/12/2358/s1, Table S1: Exact values of mRNA expression of PPARγ and PPARδ and embryos at different stages of development and quality, Table S2: *p*-values of mRNA expression of PPARγ and PPARδ and embryos at different stages of development and quality.
