# Supplementary material for: mRNA Expression and Role of PPARγ and PPARδ in Bovine Preimplantation Embryos Depending on the Quality and Developmental Stage
Source: Animals (Basel). 2020 Dec 10;10(12):2358. doi: 10.3390/ani10122358 (PMC7763280; doi:10.3390/ani10122358)
Supplement: Supplementary file 1 [file animals-10-02358-s001.pdf]

animals

MDPI

Supplementary file

Table S1. Exact values acquired in analysis of mRNA expression of PPARγ and PPARδ in bovine embryos at different stages of development and quality. Table contains exact n number and mean ± SEM for mRNA expression of PPARs in groups of early- (ECE) and late-cleaved (LCE) embryos at different stages of development (early embryo. morula and blastocyst: early. developing. expanded and hatched) and in accordance to their quality (A – good, B – moderate, C - poor quality). P – values documenting presence or lack of the significant differences between mRNA expression in ECE and LCE embryos were provided. Statistical analysis were determined by a two-way ANOVA followed by Bonferroni's multiple comparison test.

**Developmental & quality stage**		**PPARγ**					**PPARδ**				
		**ECE**		**LCE**		**p - value**	**ECE**		**LCE**		**p - value**
		**n**	**Mean ± SEM**	**n**	**Mean ± SEM**		**n**	**Mean ± SEM**	**n**	**Mean ± SEM**	
Early embryo	2-cell	4	0.01204 ± 0.005	5	0.02662 ± 0.006	0.2622	10	0.004369 ± 0.001	10	0.01420 ± 0.002	0.0039
	4-cell	5	0.01328 ± 0.005	5	0.03347 ± 0.007	0.0420	10	0.005936 ± 0.002	10	0.01166 ± 0.001	0.1814
	8-cell	3	0.01788 ± 0.004	5	0.01648 ± 0.006	0.9997	9	0.007359 ± 0.001	10	0.009885 ± 0.002	0.8633
	16-cell	5	0.003619 ± 0.001	5	0.01816 ± 0.006	0.2157	10	0.006330 ± 0.002	8	0.01494 ± 0.004	0.0231
Morula	A	2	0.0017995 ± 3.150e-005	3	0.005022 ± 0.001	0.2203	10	0.009085 ± 0.001	10	0.003772 ± 0.001	0.0594
	B	4	0.00132125 ± 1.025e-004	3	0.00307933 ± 0.001	0.5576	10	0.0079996 ± 0.002	10	0.0044645 ± 4.879e-004	0.2416
	C	5	0.0032406 ± 0.001	3	0.0121158 ± 0.002	<0.0001	10	0.0071206 ± 0.002	10	0.0122112 ± 0.02	0.0463
Early blastocyst	A	5	0.0001666 ± 5.319e-006	3	0.0004202 ± 4.908e-005	0.8306	8	0.00088 ± 1.188e-004	10	0.002175 ± 3.12e-004	0.9979
	B	4	0.001566 ± 5.576e-005	2	0.0009385 ± 4.025e-004	0.3215	10	0.002723 ± 0.001	8	0.009878 ± 0.003	0.7527
	C	7	7.561e-004 ± 8.171e-006	4	0.003131 ± 0.001	<0.0001	8	3.225e-004 ± 5.669e-007	10	0.08142 ± 0.012	<0.0001
Developing blastocyst	A	4	1.179e-004 ± 4.907e-007	3	4.077e-004 ± 1.461e-004	0.0450	10	0.001555 ± 2.828e-004	10	0.002269 ± 1.223e-004	0.2576
	B	3	4.097e-004 ± 1.233e-005	2	0.000	0.0174	10	0.001726 ± 3.114e-004	8	0.002739 ± 0.001	0.0788
	C	3	0.001 ± 3.775e-005	3	2.867e-004 ± 1.144e-004	0.0045	8	0.0007583 ± 1.051e-004	10	0.002742 ± 2.443e-004	0.0001
Expanded blastocyst	A	7	1.006e-004 ± 3.459e-005	6	0.001 ± 4.919e-005	<0.0001	13	0.0008442 ± 1.18e-004	7	0.001155 ± 1.216e-004	0.7320
	B	6	0.001 ± 6.955e-005	5	0.001 ± 1.517e-004	0.9997	13	0.001163 ± 2.254e-004	7	0.002009 ± 4.091e-004	0.0167

	C	6	3.580e004 ± 4.472e-007	6	0.001 ± 2.111e-005	0.0016	8	0.001856 ± 1.955e-004	2	0.002460 ± 4.715e-004	0.9516
Hatched blastocyst	A	7	0.001 ± 1.542e-004	6	4.167e-004 ± 7.187e-005	0.7551	21	0.001047 ± 9.047e-005	3	0.001188 ± 3.365e-004	0.9927

Table S2. P – values documenting presence or lack of the significant differences between mRNA expression of PPARγ and PPARδ in groups of early- (ECE) and late-cleaved (LCE) embryos at different stages of development (early embryo. morula and blastocyst: early. developing. expanded and hatched) and in accordance to their quality (A – good, B – moderate, C - poor quality). Statistical analysis were determined by a two-way ANOVA followed by Bonferroni's multiple comparison test.

**Developmental & quality stage**		**PPARγ**		**PPARδ**	
		**p - value**		**p - value**	
		ECE	LCE	ECE	LCE
Early embryo	2-cell vs 4-cell	0.9986	0.7913	0.9465	0.8106
	2-cell vs 8-cell	0.9133	0.5270	0.7385	0.4367
	2-cell vs 16-cell	0.7078	0.6653	0.9017	0.9947
	4-cell vs 8-cell	0.9488	0.1222	0.9621	0.9247
	4-cell vs 16-cell	0.5661	0.1865	0.9990	0.6998
	8-cell vs 16-cell	0.3570	0.9958	0.9850	0.3465
Morula	A vs B	0.9531	0.4319	0.8380	0.9517
	A vs C	0.6356	0.0010	0.5637	0.0019
	B vs C	0.3065	0.0001	0.8904	0.0021
Early blastocyst	A vs B	0.0005	0.4283	0.9710	0.5995
	A vs C	0.0877	<0.0001	0.9975	<0.0001
	B vs C	0.0242	<0.0001	0.9510	<0.0001
Developing blastocyst	A vs B	0.0364	0.0154	0.9121	0.5446
	A vs C	0.0002	0.5299	0.1826	0.5005
	B vs C	0.0293	0.0881	0.0849	>0.9999
Expanded blastocyst	A vs B	0.0022	0.5034	0.5431	0.0511
	A vs C	0.1541	0.9426	0.0024	0.1774
	B vs C	0.3678	0.2271	0.0639	0.9898
Hatched blastocyst*	4A vs 3A	0.0022	0.0361	0.7821	0.9998
	4A vs 3B	0.9987	0.5818	0.9480	0.2153
	4A vs 3C	0.4192	0.0085	0.0110	0.3074

*Expression of PPARs measured in hatched blastocysts (4) were compared with expression measured in expanded blastocyst (3) in different quality groups.

© 2020 by the author. Licensee MDPI, Basel, Switzerland. This article is an open access article distributed under the terms and conditions of the Creative Commons Attribution (CC BY) license (http://creativecommons.org/licenses/by/4.0/).
